# Supplementary material for: KEYLINK: towards a more integrative soil representation for inclusion in ecosystem scale models—II: model description, implementation and testing
Source: PeerJ. 2021 Jan 15;9:e10707. doi: 10.7717/peerj.10707 (PMC7812927; doi:10.7717/peerj.10707)
Supplement: Supplemental Information 1 [file peerj-09-10707-s001.docx]

Supplemental File S1

**KEYLINK: towards a more integrative soil representation for inclusion in ecosystem scale models. II. Model description, implementation and testing.**

**Omar Flores^*^, Gaby Deckmyn, Jorge Curiel Yuste, Mathieu Javaux, Alexei V. Uvarov, Sietse van der Linde, Bruno De Vos, Harry Vereecken, Juan José Jiménez, Olga Vindušková, Andrea Schnepf**

**Corresponding Author:**

Omar Flores

Email address: dr.flores.omar@gmail.com

**1: Review of input parameters and carbon pools**

**Respiration**

Due to the lack of the experimental data, it is mostly not possible to distinguish between (1) the standard metabolism and metabolism in the active state; (2) the ecological groups within the taxa.

To convert O_2_ consumed into carbon respiration losses, for all the animal groups it is assumed that:

(1) Respiratory quotient RQ (volumetric ratio V_CO2_/V_O2_) is 1.0, where V_O2_ – volume of oxygen consumed, V_CO2_ – volume of carbon dioxide produced; thus 1 mm^3^ O_2_ corresponds to 1 mm^3^ СO_2_. [This is a simplification, in fact RQ values can be lower (sometimes much lower); however, few realistic estimates are available. Possible corrections for some groups, i.e. more realistic RQ values, are indicated in Part 2].

(2) C_R_ = 12V_CO2_/22.4, where C_R_ – carbon respired (g); V_CO2_ – volume of CO_2_ respired (L).

Table S1.1. Respiration rates of soil invertebrates, rough estimates.

An adaptation of available data from 105 literature sources and own measurements by A.V. Uvarov.

| Group | T ^o^C | Respiration rates,  mm^3^ O_2_ g^-1^ live wt h^-1^ | Arbitrary ‘mean’, mm^3^ O_2_ g^-1^h^-1^ | Q_10_ |
| --- | --- | --- | --- | --- |
| Nematoda | 20 | 450 – 4600 | 2000 | ~ 3-4 |
| Enchytraeidae | 20 | 100 – 1500 | 500 | ~ 2-3 |
| Lumbricidae | 20 | 40 – 240 | 100 | ~ 2 |
| Isopoda (Oniscoidea) | 20 | 90 – 1600 | 300 | ~ 2.5 |
| Oribatei | 10 | 40 – 480 | 150 | ~ 3.5 |
| Oribatei | 15 | 70 – 700 | 250 | ~ 3 |
| Mesostigmata (Gamasina only) | 10 | 180 – 1600 | 500 | ~ 3-4 |
| Mesostigmata  (Gamasina, Uropodina, Trachytina) | 10 | 100 – 1600 | 400 | ~ 3 |
| Araneida | 20 | 20 – 1600 | 250 | ~ 2-3 |
| Diplopoda | 20 | 20 – 900 | 150 | ~ 2 |
| Chilopoda | 20 | 100 – 800 | 250 | ~ 3 |
| Collembola | 10 | 50 – 1300 | 400 | ~ 3 |
| Collembola | 15 | 50 – 2700 | 600 | ~ 3 |
| Carabidae, imago | 15 | 80 – 1300 | 350 | ~ 3 |
| Staphylinidae, imago | 15 | 150 – 850 | 400 | ~ 3-4 |
| Coleoptera, larvae | 15 | 70 –2500 | 550 | ~ 3 |
| Coleoptera, larvae | 20 | 80 –2600 | 750 | ~ 3 |
| Diptera larvae | 20 | 200 – 2200 | 800 | ~ 2-3 |

**C:N ratios**

C:N ratios are an important input for the model. Data can be readily found for many soil animal species. The C:N ratio of root herbivores has been reported to be lower than their food sources. The average C to N ratio of microfauna is about 10 (range between 7.5-12, Anderson *et al*., 1981; Hunt *et al*., 1987). Soil arthropods typically have a C content of about 50% and a N content around 10%, leading to a C:N ratio of about 5. According to Hunt *et al*. (1987), Prostigmata have a C:N = 8. Based on information provided in Pokarzhevskii *et al*. (2003), the C:N ratio of adult Scarabaeid beetles is 5.43 and of Diptera larvae C:N = 4.46. No information is given for the Symphyla, but their relatives, Chilopoda, have a C:N = 4.89. For fungi and bacteria a wide range of values have been found but in general bacteria have a lower C:N ratio. Chertov *et al*. (2017) use an empirical model to calculate local C:N ratio based on the SOM C:N. *Ferris et al*. (1997) provide C:N values for bacterial feeding nematodes, i.e. 5.9, and for the populations of *Escherichia coli* they grew on, i.e. 4.1.

**Table S1.2. Carbon pools in the KEYLINK model.**

Pools of the soil food web represent different functional groups.

|  | Symbol | C pool |
| --- | --- | --- |
| 1 | B_b_ | bacterial biomass |
| 2 | B_f_ | fungal biomass |
| 3 | B_myc_ | mycorrhizal biomass |
| 4 | B_bvores_ | biomass bacterivores |
| 5 | B_fvores_ | biomass fungivores |
| 6 | B_det_ | biomass detritivores |
| 7 | B_eng_ | biomass engineers |
| 8 | B_hvores_ | biomass herbivores |
| 9 | B_pred_ | biomass predators |
| 10 | L_surf_ | aboveground litter originating from trees |
| 11 | SOM | total soil organic matter |
| 12 | B_root_ | biomass roots |
| 13 | R | respiration (CO_2_) |

**References**

Anderson, R. V., Coleman, D. C., Cole, C. V., and Elliott, E. T. (1981). Effect of the nematodes Acrobeloides sp. and Mesodiplogaster lheritieri on substrate utilization and nitrogen and phosphorous mineralization in soil. *Ecology, 62*(3), 549-555.

Chertov, O., Komarov, A., Shaw, C., Bykhovets, S., Frolov, P., Shanin, V., et al. (2017). Romul_Hum - A model of soil organic matter formation coupling with soil biota activity. II. Parameterisation of the soil food web biota activity. *Ecological Modelling, 345*, 140-149.

Ferris, H., Venette, R. C., and Lau, S. S. (1997). Population energetics of bacterial-feeding nematodes: carbon and nitrogen budgets. *Soil Biology and Biochemistry, 29*(8), 1183-1194.

Hunt, H. W., Coleman, D. C., Ingham, E. R., Ingham, R. E., Elliott, E. T., Moore, J. C, et al. (1987). The detrital food web in a shortgrass prairie. *Biology and Fertility of Soils, 3*(1-2), 57-68.

Pokarzhevskii, A. D., van Straalen, N. M., Zaboev, D. P., & Zaitsev, A. S. (2003). Microbial links and element flows in nested detrital food-webs. *Pedobiologia, 47*(3), 213-224.
