# Supplementary material for: KEYLINK: towards a more integrative soil representation for inclusion in ecosystem scale models—II: model description, implementation and testing
Source: PeerJ. 2021 Jan 15;9:e10707. doi: 10.7717/peerj.10707 (PMC7812927; doi:10.7717/peerj.10707)
Supplement: Supplemental Information 2 [file peerj-09-10707-s002.docx]

Supplemental File S2

**KEYLINK: towards a more integrative soil representation for inclusion in ecosystem scale models. II. Model description, implementation and testing.**

**Omar Flores^*^, Gaby Deckmyn, Jorge Curiel Yuste, Mathieu Javaux, Alexei V. Uvarov, Sietse van der Linde, Bruno De Vos, Harry Vereecken, Juan José Jiménez, Olga Vindušková, Andrea Schnepf**

**Corresponding Author:**

Omar Flores

Email address: dr.flores.omar@gmail.com

**2: Input parameters for Brasschaat run**

Each table represent an input file (specific names of each text file are given between brackets).

**Table S2.1.** **Initial C in each pool ("KL_initC_pools").**

For the scenario excluding predators (“Bpred 0”), the initial biomass of predators was set to 0.

| Pool | Initial biomass (g C m^-3^) |
| --- | --- |
| Bacteria | 15.1 |
| Fungi | 15.1 |
| Mycorrhiza | 160 |
| Bacterivores | 0.1 |
| Fungivores | 0.8 |
| Detritivores | 0.6 |
| Engineers | 0.2 |
| Herbivores | 0.2 |
| Predators | 0.4 |
| Litter | 2680 |
| SOM | 11470 |
| Roots | 320 |

**Table S2.2.** **Faunal parameter values used for the Brasschaat run ("KL_FaunalParams").**

Each column is a food web pool: bacteria (bact), fungi (fung), mycorrhiza (myc), bacterivores (Bvores), fungivores (Fvores), detritivores (detrVor), engineers (eng), herbivores (herbv) and predators (pred). Each row is a parameter vector: g_max_ and Ks used for the showed simulations, death and resp are the rates of death and respiration, faeces is the equivalent fraction of growth that is transformed to faeces, C:N (ratio) and recalcitrance (recalc) of each pool, pmC:N and pmRec are the sensibility parameters to C:N and recalcitrance (for equations 23-26 in the paper), the minimum (min), optimum (opt) and maximum (max) temperatures (T) for the growth of each population, and the Q10.

|  | Units | Bact | Fung | Myc | Bvores | Fvores | Detrvor | Eng | Herbv | Pred |
| --- | --- | --- | --- | --- | --- | --- | --- | --- | --- | --- |
| g_max_ | gC gC^-1^ day^-1^ | 1.24 | 0.6 | 0.44 | 1.4 | 0.8 | 0.178 | 0.109 | 0.135 | 0.096 |
| Ks | g m^-3^ | 5500 | 5500 | 5500 | 7.5 | 7.5 | 5500 | 5500 | 160 | 2 |
| death | gC gC^-1^ day^-1^ | 0.05 | 0.01 | 0.04 | 0.02 | 0.02 | 0.0013 | 0.0065 | 0.005 | 0.005 |
| resp | gC gC^-1^ day^-1^ | 0.03 | 0.03 | 0.03 | 0.02 | 0.02 | 0.01 | 0.01 | 0.01 | 0.01 |
| faeces |  | 0 | 0 | 0 | 0 | 0 | 0.3 | 0.3 | 0.3 | 0.3 |
| C:N |  | 4 | 8 | 9 | 6 | 9 | 5 | 5 | 8 | 8 |
| recalc |  | 0.2 | 0.4 | 0.5 | 0.6 | 0.8 | 0.9 | 0.9 | 0.1 | 0.1 |
| pmC:N |  | 0.8 | 0.3 | 0.3 | 0 | 0 | 0 | 0 | 0 | 0 |
| pmRec |  | 0.9 | 0.75 | 0.8 | 0.8 | 0.8 | 0.5 | 0.5 | 0.8 | 0.8 |
| T min | ºC | 0 | 0 | 0 | 0 | 0 | 0 | 0 | 0 | 0 |
| T opt | ºC | 25 | 25 | 25 | 25 | 25 | 15 | 15 | 15 | 15 |
| T max | ºC | 40 | 40 | 40 | 40 | 40 | 40 | 40 | 40 | 40 |
| Q10 |  | 2.5 | 2.5 | 2.5 | 2.5 | 2.5 | 2.5 | 2.5 | 2.5 | 2.5 |

**Table S2.3.** **Monthly average temperatures and total sunlight hours in the simulated site ("KL_climateParams").**

| Month | Jan | Feb | March | April | May | Jun | July | Aug | Sept | Oct | Nov | Dec |
| --- | --- | --- | --- | --- | --- | --- | --- | --- | --- | --- | --- | --- |
| Average temperature (ºC) | 3.3 | 3.7 | 6.8 | 9.8 | 13.6 | 16.2 | 18.4 | 18.0 | 14.9 | 11.1 | 6.8 | 3.9 |
| Monthly total sunlight hours | 59 | 77 | 114 | 159 | 191 | 188 | 201 | 190 | 143 | 113 | 66 | 45 |

**Table S2.4.** **Parameters of soil characteristics ("KL_initSoil").**

Litter CN ratio here refers to initial litter quality in the litter pool. pH was set to 5.9 for the alternative scenario “pH 5.9”. And for the alternative scenario “clay 15%”, the volumes of the five pore classes were the following (respectively): 142, 80, 80, 200, 6.

| Variable | Units | Value |
| --- | --- | --- |
| Depth of soil layer | m | 1 |
| Bulk density | kg m^-3^ | 1463 |
| alpha (van Genuchten) | kPa^-1^ | 1.2 |
| n (van Genuchten) |  | 1.7 |
| m (van Genuchten) |  | 0.3 |
| Ksat (van Genuchten) |  | 10 |
| pH |  | 3.9 |
| Initial litter CN ratio |  | 38.6 |
| Initial SOM CN ratio |  | 18 |
| Drainmax | mm day^-1^ | 7 |
| Volume of inaccessible pores | l m^-3^ | 45 |
| Volume of bacterial pores | l m^-3^ | 37 |
| Volume of micropores | l m^-3^ | 37 |
| Volume of mesopores | l m^-3^ | 200 |
| Volume of macropores | l m^-3^ | 6 |

**Table S2.5.** **Parameters for engineers and detritivores activity ("KL_engineerParams").**

Rows show ratio of pore volume to engineer biomass (VE_ratio_), fraction of volume that is made by extra porosity (f_PV_), the daily turnover of burrows, maximum burrow volume (PV_Bmax_), fraction of litter fragmentation, sensitivity of % faeces to CN ratio (m_faec_) for engineers and detritivores, bioturbation and litter moved as the daily amount of SOM and litter (respectively) that engineers bring to deeper layers.

| Variable | Units | Value |
| --- | --- | --- |
| VE_ratio_ | l g^-1^ | 1 |
| f_PV_ | 0.5 | 0.5 |
| Turnover of burrows | day^-1^ | 0.01 |
| PV_Bmax_ | l m^-3^ | 25 |
| Litter fragmentation | day^-1^ | 0.05 |
| m_faec_ for engineers |  | 0.2 |
| m_faec_ for detritivores |  | 0.3 |
| Bioturbation | % SOM g C_Eng_^-1^ day^-1^ | 0.05 |
| Litter moved by engineers | % litter g C_Eng_^-1^ day^-1^ | 0.01 |

**Table S2.6**. **Model run options ("KL_runparams").**

The C fraction of N from mycorrhiza ("myc") to plants is the fraction (0-1) of the N input to mycorrhiza that they receive from plants (and the rest comes from the soil). Litter CN ratio here refers to the litter quality of input litter added daily to the litter pool, which was set to 40 for the alternative scenario “CNlit 40”. And the recalcitrance of litter was set to 20 for the alternative scenario “rec 20%”.

| Variable | Units | Value |
| --- | --- | --- |
| Simulation time | days | 3653 |
| Initial soil water | % | 100 |
| Initial mineral N | g N m^-3^ | 5 |
| Root growth | g C m^-3^ day^-1^ | 0.575 |
| Root turnover | g C m^-3^ day^-1^ | 0.003 |
| Litter input | g C m^-3^ day^-1^ | 1.32 |
| Litter CN ratio |  | 60.3 |
| Recalcitrance of litter | % | 40 |
| C input to mycorrhiza | g C m^-3^ day^-1^ | 0.54 |
| Fraction N from myc to plants |  | 0.9 |
| Fraction effective evapotranspiration |  | 0.7 |

Model outputs

After each simulation, together with graphs automatically created by the model showing daily variations in all C pools, soil water content and soil porosity, KEYLINK creates a new text file named "keylinkoutput" in the same folder, and when it already exists, a new simulation overwrites that file, so we recommend to copy it in other folder or to change its name before every new simulation in order to keep all results. This text file has a row for each simulated day, and C pools biomass and some C fluxes (g C m^-3^) in 21 columns, in the following order: (1) bacteria, (2) fungi, (3) mycorrhiza, (4) bacterivores, (5) fungivores, (6) detritivores, (7) engineers, (8) herbivores, (9) predators, (10) litter, (11) SOM, (12) roots, (13) cumulative CO2 emissions, (14) daily respiration from bacteria, (15) from fungi and (16) from mycorrhiza, (17) C flux from SOM to bacteria, (18) C flux from litter to bacteria, (19) total SOM eaten, (20) total litter eaten, and (21) litter eaten by engineers.
